# Genetic Diversity of Beet Necrotic Yellow Vein Virus in Serbia: First Detection of J-Type RNA5 and P25 Tetrad Associated with Resistance-Breaking Isolates

**DOI:** 10.3390/microorganisms14092030

**Published:** 2026-09-11

**Authors:** Anja Milosavljević, Ivana Stanković, Danijela Ristić, Tatjana Popović Milovanović, Katarina Zečević, Jelena Đukanović, Nenad Trkulja

**Affiliations:** 1 Research and Development Center Sunoko, Ljukovo, 22321 Inđija, Serbia; 2 University of Belgrade, Faculty of Agriculture, Nemanjina 6, 11080 Belgrade, Serbia; ivana.stankovic@agrif.bg.ac.rs (I.S.); katarina.zecevic@agrif.bg.ac.rs (K.Z.); 3 Institute for Plant Protection and Environment, Teodora Drajzera 9, 11040 Belgrade, Serbia; risticdaca@yahoo.com (D.R.); tanjaizbis@gmail.com (T.P.M.);

**Keywords:** sugar beet, rhizomania, BNYVV, RNA3, RNA5, phylogenetic analysis, resistance-breaking isolates

## Abstract

Beet necrotic yellow vein virus (BNYVV), the causal agent of rhizomania, is a major threat to sugar beet production worldwide. In Serbia, during a three-year monitoring period (2023–2025) conducted across 31 localities (72 fields) representing the main sugar beet production areas, BNYVV was detected in 26.5% of analyzed samples. Of the 88 ELISA-positive samples, 60 originated from cultivars carrying the *Rz1*, whereas 28 were obtained from cultivars carrying the combined *Rz1+Rz2* resistance genes. This study presents the first analysis of the genetic diversity and population structure of BNYVV in Serbia, based on P25 tetrad variability and RNA5 sequence analysis. Sequence analysis revealed five distinct P25 tetrad variants, with the ALHG tetrad being the most prevalent. Moreover, in one locality in the Bačka region, the VHHG variant, previously associated with *Rz1*-resistance-breaking isolates, was also detected. Phylogenetic analysis based on p25 sequences grouped all Serbian isolates within the P25-I cluster, corresponding to the A-type virus population, and showed close relationship with European isolates, particularly those from Italy. Importantly, RNA5 was detected in all selected isolates, representing the first report of RNA5 in Serbia. The results of the p26sequence analysis revealed high conservation among Serbian isolates, which clustered within the J-type in phylogenetic analysis. Overall, the results demonstrate that Serbian BNYVV populations are genetically diverse and include variants associated with *Rz1*-resistance breaking. These findings highlight the need for continuous molecular surveillance to monitor the emergence and spread of virulent strains and to support effective disease management strategies in sugar beet cultivation.

## 1. Introduction

Sugar beet (*Beta vulgaris* L.; family *Chenopodiaceae*) is a major industrial crop grown for its high sucrose content in the root and is the primary source of sugar in temperate regions. Globally, the largest producers include European countries such as Germany, France, Poland, and the UK, as well as the USA and Russia [1]. Sugar beet is also a key contributor to the Serbian agricultural sector. Production is concentrated mainly in the Province of Vojvodina, where favorable agroclimatic conditions enable stable and efficient production. The Bačka and Srem districts are the principal production areas, accounting for more than 60% of the total cultivated area, while other regions contribute substantially less to national production [2].

Despite its economic importance, sugar beet cultivation is highly demanding, especially in terms of plant protection, as it requires more intensive management compared to most other field crops. Rhizomania, a viral disease caused by beet necrotic yellow vein virus (BNYVV), belonging to species *Benyvirus necrobetae*, a member of the genus *Benyvirus* within the family *Benyviridae*, is a globally distributed disease associated with significant losses in both root yield and sugar content [3]. Typical symptoms on the taproot include pronounced proliferation of lateral roots, overall root size reduction, and necrosis of vascular tissues. Although chlorosis and necrosis of leaf veins may develop following systemic infection, such foliar symptoms are rarely observed under field conditions. The soil-borne plasmodiophoromycete *Polymyxa betae* acts as the vector of BNYVV [4,5,6]. The persistence of the virus in soil is ensured by its association with long-lived resting spores of the vector, enabling survival for long periods.

The genome of BNYVV consists of four to five positive-sense single-stranded RNA molecules, each characterized by a 5′ m7G cap structure and a 3′ polyA tract [4,6,7]. RNA1 and RNA2 harbor “housekeeping” genes that are involved in virus replication, assembly, intercellular movement, and suppression of post-transcriptional gene silencing, while RNA3, RNA4, and RNA5 are critical for pathogenicity, systemic movement, and transmission by the vector, thereby contributing to the full expression of rhizomania symptoms [8]. RNA1 contains one open reading frame (ORF) and encodes an RNA-dependent RNA polymerase with conserved domains typical of methyltransferase, helicase, and papain-like protease activities [6]. RNA2 contains six ORFs encoding the coat protein (CP), a CP-readthrough (CP-RT) protein required for transmission by the vector, the triple gene block proteins (TGB1–TGB3) that mediate cell-to-cell movement, and cysteine-rich protein p14, a viral suppressor of RNA silencing [4]. RNA3 encodes the P25 protein, responsible for the expression of rhizomania symptoms and the resistance-breaking ability of the virus [9,10], while RNA4 encodes the p31 protein, a multifunctional protein implicated in efficient vector transmission, enhanced symptom expression, and root-specific silencing suppression [11]. Certain BNYVV isolates possess an additional RNA5 with one ORF encoding the P26 protein, another pathogenicity factor associated with a synergistic effect on disease development and transmission; these isolates are frequently linked to highly aggressive forms of rhizomania disease [12,13].

Previously, two major types of BNYVV, designated as types A and B, were described based on coat protein gene sequences [4,6]. A third, less common type, designated as the P-type, was reported from France by Koenig et al. [14], and contains an additional RNA5 segment. Isolates containing RNA5 occur in limited areas of Europe but are widely distributed in China and Japan. Asian strains with RNA5 were designated as the J-type [13,15,16,17]. J-type isolates possess two deletions at amino acid positions 77 and 227–229 in P26 compared to the P-type [15]. Previous studies suggested that the P-type RNA5 was geographically restricted to France, the United Kingdom, and Kazakhstan [12,16,18], while the J-type mainly occurs in Japan, China, and a single field in Germany [17,19]. However, recent investigations conducted in Turkey [20] and several European countries [21] have challenged this assumption, suggesting a wider geographic distribution of J-type than previously assumed. Phylogenetic analyses have grouped RNA5 sequences into three distinct clusters, with groups I and II associated with Asian isolates (J-type), while group III corresponds to the P-type found in French populations [13].

Control of rhizomania relies primarily on cultivating sugar beet resistant varieties, which reduces yield losses caused by disease development. The *Rz1* gene is the main source of resistance against BNYVV and is widely incorporated into most commercial sugar beet cultivars [22]. This gene was identified in 1983 and subsequently introduced in all sugar beet cultivars [6,23]. However, intensive selection pressure on *Rz1* has led to the emergence of resistance-breaking (RB) variants. RB isolates were first reported in California [24], and subsequently in other parts of the United States [25], as well as in several countries across Europe and the Middle East [5,10,20,21]. Sequence analysis of the *p25* gene revealed that the ability to overcome *Rz1*-mediated resistance is associated with amino acid substitutions at positions 67–70, with specific tetrad variants linked to the occurrence of RB isolates [21,25,26,27]. *Rz2* represents the second major resistance gene incorporated into sugar beet breeding programs. It operates through a resistance mechanism distinct from that of *Rz1* and remains highly effective in areas where BNYVV populations have overcome *Rz1*-mediated resistance. Notably, *Rz2* was the first rhizomania resistance gene to be molecularly cloned, and the BNYVV triple gene block I (TGB1) was subsequently identified as the corresponding viral avirulence determinant [28]. In addition, the presence of the RNA5 segment has also been shown to contribute to resistance breaking [29,30], but this effect seems to be independent of the P25 tetrad variant and therefore based on a different mechanism [21].

In recent years, sugar beet growers in Serbia have increasingly reported rhizomania symptoms in resistant cultivars, as well as changes in sugar and nitrogen content in these cultivars, indicating the possible presence of resistance-breaking BNYVV populations in the country. In this study, the *p25* and *p26* genes were amplified and sequenced from isolates collected from infected sugar beet roots to perform molecular characterization and assess the resistance-breaking potential of the analyzed populations in Serbia. Sugar beet cultivars currently cultivated in Serbia predominantly carry the *Rz1* resistance gene, whereas only a limited proportion possess the combined *Rz1*+*Rz2* resistance genes.

## 2. Materials and Methods

### 2.1. Sampling and Isolates Collection

**Figure 1:**
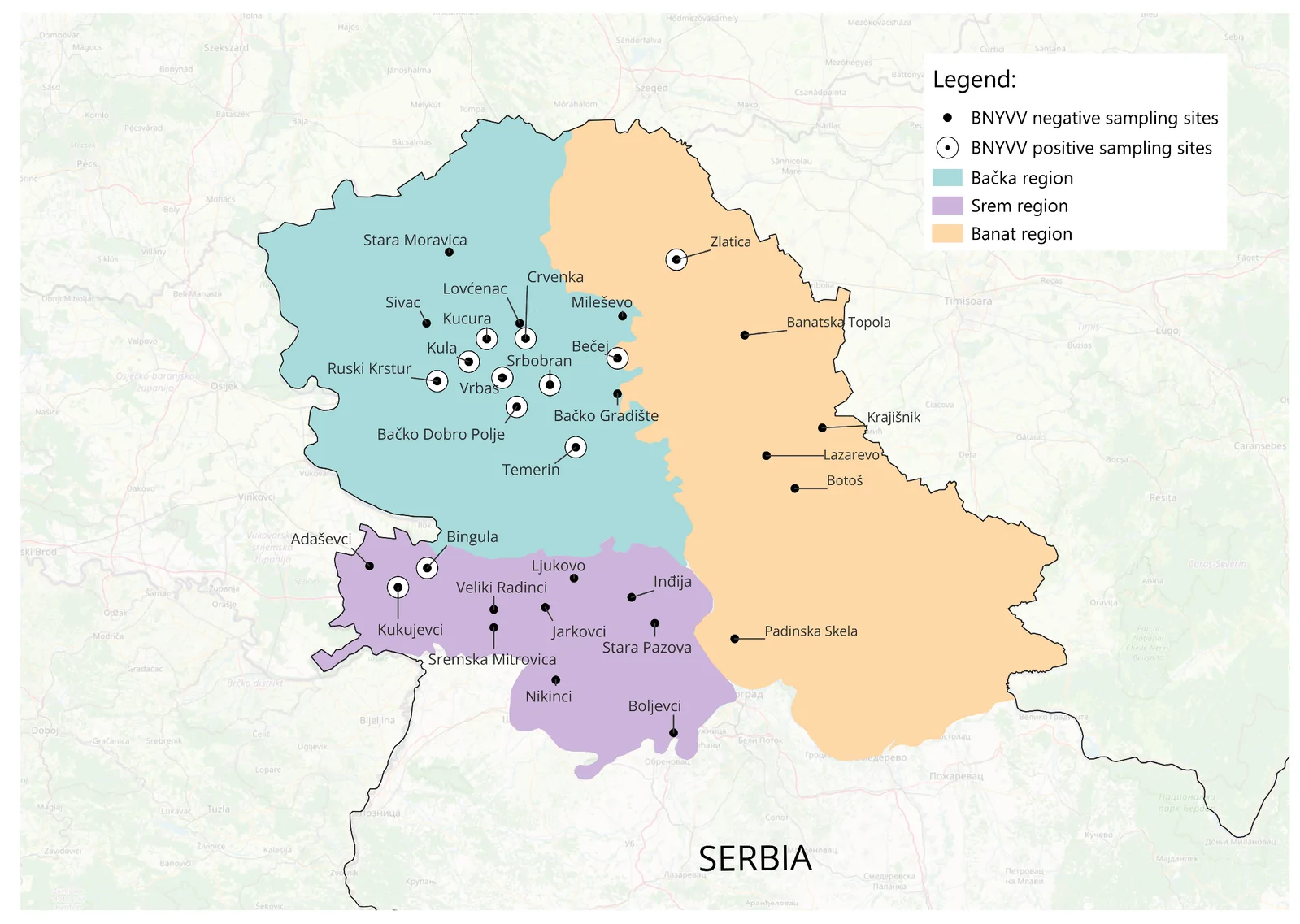
Map showing the sugar beet sampling sites in the Vojvodina region, Serbia, during 2023–2025.

To identify factors associated with production problems in sugar beet cultivars in Serbia, root samples were collected from *Rz1* (in total 24) and *Rz1+Rz2* (in total 6) resistant cultivars grown under natural infection conditions at 31 localities from 72 fields with a long history of sugar beet cultivation. Sampling took place throughout Vojvodina as the main sugar beet-producing region, during a three-year survey conducted from 2023 to 2025 (Figure 1, Table 1). Root samples were collected from each field from sugar beet plants exhibiting symptoms characteristic of rhizomania (yellowing of leaves and proliferation of lateral roots-bearded roots and reduced root size. Each sample represents one individual root. The samples were stored at −80 °C until further serological and molecular analyses were performed.

### 2.2. Serological Testing

Samples of fibrous roots were screened for the presence of BNYVV, utilizing the double-antibody sandwich (DAS)-ELISA kits (Anti BNYVV IgG-Bioreba AG, Reinach, Switzerland) as described by Clark and Adams [31]. The tests were performed in polystyrene microplates (Thermo Fisher Scientific, Roskilde, Denmark), according to the manufacturer’s instructions. Commercial positive and negative controls (Bioreba AG, Reinach, Switzerland) were included in each test. Absorbance at 405 nm was measured using an ELISA reader (CLARIOstar Plus, BMG Labtech, Ortenau, Germany) after 1 or 2 h of incubation with p-nitrophenyl phosphate at room temperature in the dark. Samples were considered positive when the average optical density (OD) value was at least two times higher than that of the virus-free control samples.

### 2.3. RNA Extraction and RT-PCR

Total RNAs were extracted using the RNeasy Plant Mini Kit (Qiagen, Hilden, Germany) from 100 mg of frozen root tissue. A total of 17 representative ELISA-positive samples were selected for analysis, representing different regions and/or sampling years, prioritizing those originating from sites with the highest disease incidence. In total 15 isolates were originated from *Rz1*, while 2 other isolates (24/21 and 24/96) were originated from the *Rz1*+*Rz2* resistant cultivar obtained from the same sugar beet field, but at two different sampling period but in the same year (2024). A reverse transcriptase-PCR (RT-PCR) was performed using *p25* open reading frame (ORF) specific primers BNYVV-3F (5′-ACCGACCAAATCCAAGCGAG-3′) and BNYVV-3R (5′-CGCTACTGCACACTCTTTACCA-3′) to amplify a 1330 bp fragment of BNYVV RNA3 [32]. The presence of RNA5 was assayed by amplifying a 530 bp fragment of the *p26* ORF using specific primers BNYVV-5F1 (5′-GATATGGCATATAGCGACG-3′) and BNYVV-5R1 (5′-GGTCGTTGCCAAAATCTC-3′) [12]. A tissue sample from healthy sugar beet roots was used as a negative control in RNA extraction and RT-PCR assays. RT-PCR was carried out using the One-Step RT-PCR kit (Qiagen) according to the manufacturer’s instructions.

The RT-PCR reaction mixture included 5 μL 5× Qiagen OneStep RT-PCR buffer (containing 12.5 mM MgCl_2_), 400 μM each of the four dNTPs, 1 μL of RT-PCR enzyme mix, 0.6 μM each primer and 1 μL extracted RNA in a final volume of 25 μL. Amplifications were performed in an Eppendorf thermal cycler Mastercycler nexus GSX1 (Eppendorf SE, Hamburg, Germany). Reverse transcription was performed at 50 °C for 30 min, followed by an initial denaturation step at 95 °C for 15 min. PCR amplification was conducted using primer-specific cycling conditions and was completed with a final extension at 72 °C for 10 min. For the BNYVV-3F/BNYVV-3R primer pair, amplification consisted of 35 cycles of denaturation at 94 °C for 1 min, annealing at 55 °C for 2 min and elongation at 72 °C for 2 min, while PCR amplification for BNYVV-5F1/BNYVV-5R1 primers was performed in 36 cycles of 96 °C for 45 s for denaturation, 52 °C for 1 min for annealing, and 72 °C for 1 min for elongation. Amplified products were analyzed by 1% agarose gel electrophoresis, stained with Midori green dye (Bulldog Bio Inc., Portsmouth, NH, USA) and visualized with a UV transilluminator (Vilber Lourmat Deutschland GmbH,, Eberhardzell, Germany).

### 2.4. Sequencing and Phylogenetic Analysis

After successful amplification of *p25* and *p26* genes, RT-PCR products were submitted for both purification and bidirectional sequencing (Macrogen Europe B.V., Amsterdam, The Netherlands) using the same primers as in the RT-PCR reactions. The obtained sequences were analyzed using FinchTV and compared using the CLUSTAL W program [33] integrated within the MEGA X software (V 10.2.4) package [32], after which a consensus sequence was generated for each selected isolate. Each p25and p26 consensus sequences was verified by BLASTn (v 2.17.0) and deposited in the GenBank database under the accession numbers (Table 2). The obtained sequences were compared with each other by calculating nucleotide (nt) and deduced amino acid (aa) identities using the p-distance model implemented in MEGA X software [34].

The deduced P25 amino acid sequences were further analyzed for tetrad variants at amino acid positions 67–70 that have previously been reported in association with resistance-breaking ability in BNYVV. The presence of these variants was used as molecular markers potentially associated with RB capacity. However, the RB phenotype of the analyzed isolates was not experimentally confirmed by biological assays using resistant sugar beet genotypes.

To assess phylogenetic relationships, Serbian p25and p26 sequences were aligned with representative sequences retrieved from the GenBank database (Supplementary Tables S1 and S2, respectively). Phylogenetic trees were reconstructed using the maximum likelihood (ML) method implemented in MEGA X with 1000 bootstrap replicates and the Hasegawa–Kishino–Yano nucleotide substitution model plus Gamma distribution (HKY + G), selected as the best-fit model according to the Bayesian Information Criterion (BIC). All branches with bootstrap support of less than 70% were collapsed.

## 3. Results

### 3.1. BNYVV Detection and Occurrence in Sugar Beet

**Figure 2:**
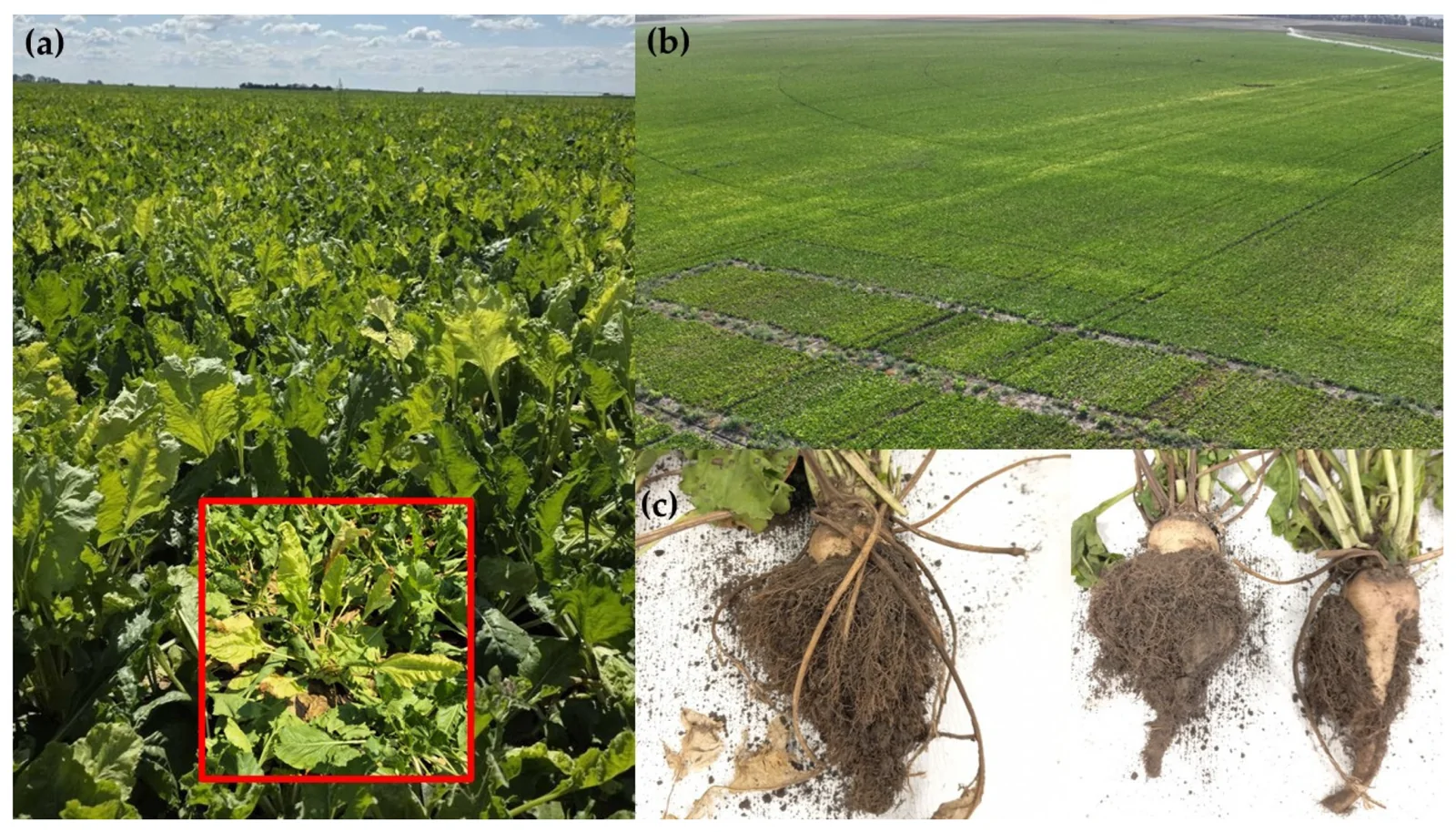
Rhizomania symptoms observed in Serbian sugar beet fields: (**a**) patchy distribution of leaf yellowing (the red square indicates the inset); (**b**) distribution of rhizomania-induced leaf yellowing in Serbian fields recorded by drone; and (**c**) taproot bearding.

Symptoms of rhizomania observed in the surveyed sugar beet fields in Vojvodina included plant stunting, pale green discoloration to yellowing of the leaves, and characteristic root symptoms (Figure 2). The affected roots exhibited extensive proliferation of lateral roots (root bearding), reduced root size, and brown vascular rings. In some fields, symptomatic plants occurred in distinct patches, whereas in others they were scattered individually throughout the field.

During a three-year survey period covering 72 sugar beet fields (31 localities) in the Vojvodina Province of Serbia, a total of 332 samples of root were collected and tested (Table 1). The presence of BNYVV was detected by ELISA in 88 sugar beet samples, indicating an overall incidence of 26.5% (Table 1). During 2023 a total count of collected samples was 157, with positive BNYVV incidence value of 19.4% in Bačka, 9.7% in Srem and 7.7% in Banat region. In 2024, 127 samples were collected, with the highest incidence of BNYVV recorded in Srem (69.6%), followed by Bačka (35.2%), whereas the presence of the virus was not detected in Banat. In 2025, a total of 48 samples were collected; a high incidence of BNYVV was recorded in Bačka (48.5%), while the virus was not detected in either Banat or Srem.

**Table 1:** Occurrence of BNYVV in sugar beet grown in the Vojvodina Province of Serbia during 2023–2025 (confirmed by ELISA test).

Region	Locality	Year	Number of Fields	Number of Tested Samples	Number of ELISA-Positive Samples (%)
Bačka	Bačko Dobro Polje	2023	6	36	16 (44.4)
		2024	3	35	10 (28.6)
		2025	2	7	6 (85.7)
	Kula	2023	4	8	3 (37.5)
		2024	1	4	4 (100)
		2025	1	5	0
	Crvenka	2023	1	2	1 (50.0)
		2025	3	12	5 (41.7)
	Bečej	2023	7	53	0
		2024	2	14	12 (85.7)
		2025	1	2	0
	Srbobran	2023	1	1	0
		2025	1	2	2 (100.0)
	Sivac	2023	2	2	0
		2025	2	2	0
	Stara Moravica	2023	1	1	0
		2024	1	1	0
	Kucura	2024	1	6	1 (16.7)
	Temerin	2024	1	5	1 (20.0)
	Bačko Gradište	2024	1	2	0
	Lovćenac	2024	1	2	0
	Mileševo	2024	1	10	0
	Ruski Krstur	2024	1	6	3 (50.0)
	Vrbas	2024	1	3	0
		2025	1	3	3 (100.0)
Banat	Banatska Topola	2023	1	2	0
	Krajišnik	2023	1	2	0
	Zlatica	2023	3	6	1 (16.7)
	Padinska Skela	2023	1	3	0
		2025	1	5	0
	Botoš	2024	1	11	0
	Lazarevo	2024	1	5	0
		2025	1	8	0
Srem	Sremska Mitrovica	2023	1	2	0
	Stara Pazova	2023	2	8	0
	Inđija	2023	1	3	0
	Bingula	2024	1	10	5 (50.0)
		2023	1	4	4 (100.0)
	Boljevci	2023	1	12	0
	Nikinci	2023	1	2	0
	Jarkovci	2023	1	8	0
	Veliki Radinci	2025	1	2	0
		2023	1	1	0
	Ljukovo	2023	1	1	0
	Adaševci	2024	1	3	3 (100.0)
	Kukujevci	2024	2	10	8 (80.0)
	Total		72	332	88 (26.5)

Over the three-year sampling period, a total of 78 samples were collected from sugar beet cultivars carrying the *Rz1*+*Rz2* resistance genes. Of these, 54 samples were collected in 2023, with 11 testing positive for BNYVV by ELISA; 15 samples were collected in 2024, of which 12 were ELISA-positive; and nine samples were collected in 2025, of which five tested positive. During the same period, a total of 254 samples were collected from cultivars carrying the *Rz1* resistance gene, of which 60 tested positive for BNYVV by ELISA. In 2023, 16 of the 103 collected samples were ELISA-positive; in 2024, 33 of 112 samples tested positive; and in 2025, 11 of the 39 collected samples were positive.

### 3.2. Molecular Characterization of Serbian BNYVV Population

**Figure 3:**
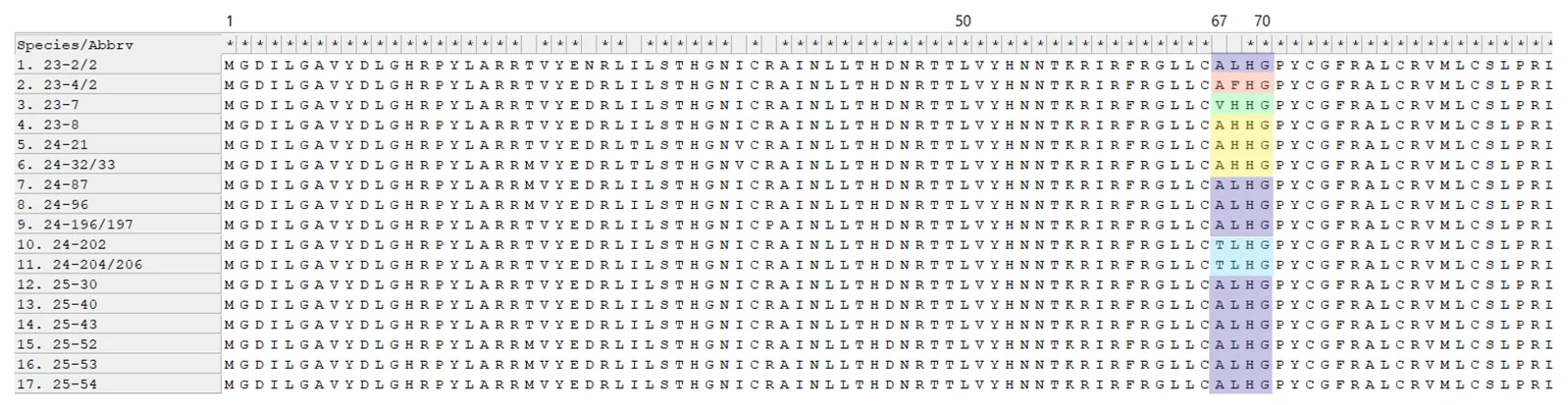
Multiple sequence alignment of P25 protein amino acid sequences from Serbian beet necrotic yellow vein virus isolates. Tetrad variants at amino acid positions 67–70 are highlighted in different colors according to their amino acid composition. The asterisk (*) denotes conserved amino acid position i.e., the same amino acid is present in all Serbian p25 sequences.

Following successful amplification of the *p25* gene fragment in all 17 selected isolates (Supplementray Figure S1), the PCR products were subjected to bidirectional sequencing. The genetic diversity of the Serbian BNYVV isolates was characterized by sequence diversity and tetrad variability at amino acid positions 67–70 of the P25 protein. The *p25* gene sequence obtained in this study showed high nucleotide and amino acid similarity, ranging from 98.9% to 100% and 97.7% to 100%, respectively. Nevertheless, five distinct tetrad variants were identified. The ALHG motif was predominant, occurring in 10 isolates, followed by AHHG in three isolates, TLHG in two isolates, and AFHG and VHHG, each detected in a single isolate (Table 2, Figure 3). No clear correlation was observed between tetrad distribution and geographic origin. Notably, the VHHG tetrad, previously associated with *Rz1*-resistance-breaking strains, was detected only in one isolate from the Bačka region.

**Table 2:** GenBank database accession numbers, diversity of RNA3 tetrad varaiants and RNA5 type in Serbian isolates of beet necrotic yellow vein virus.

Isolate Code	Year	Locality	Region	P25 Tetrad (aa Residues 67–70)	RNA5 Type	GenBank Database Accession Number	
						*p25* Gene	*p26* Gene
23-2/2	2023	Bačko Dobro Polje	Bačka	ALHG	J	PZ656453	PZ656470
23-7	2023	Bačko Dobro Polje	Bačka	VHHG	J	PZ656455	PZ656472
23-8	2023	Bačko Dobro Polje	Bačka	AHHG	J	PZ656456	PZ656473
24-87	2024	Bačko Dobro Polje	Bačka	ALHG	J	PZ656459	PZ656476
24-21	2024	Bečej	Bačka	AHHG	J	PZ656457	PZ656474
24-96	2024	Bečej	Bačka	ALHG	J	PZ656460	PZ656477
25-30	2025	Srbobran	Bačka	ALHG	J	PZ656464	PZ656481
25-40	2025	Vrbas	Bačka	ALHG	J	PZ656465	PZ656482
25-43	2025	Vrbas	Bačka	ALHG	J	PZ656466	PZ656483
23-4/2	2023	Kula	Bačka	AFHG	J	PZ656454	PZ656471
24-196/197	2024	Kula	Bačka	ALHG	J	PZ656461	PZ656478
25-52	2025	Crvenka	Bačka	ALHG	J	PZ656467	PZ656484
25-53	2025	Crvenka	Bačka	ALHG	J	PZ656468	PZ656485
25-54	2025	Crvenka	Bačka	ALHG	J	PZ656469	PZ656486
24-32/33	2024	Kukujevci	Srem	AHHG	J	PZ656458	PZ656475
24-202	2024	Kukujevci	Srem	TLHG	J	PZ656462	PZ656479
24-204/206	2024	Kukujevci	Srem	TLHG	J	PZ656463	PZ656480

**Figure 4:**
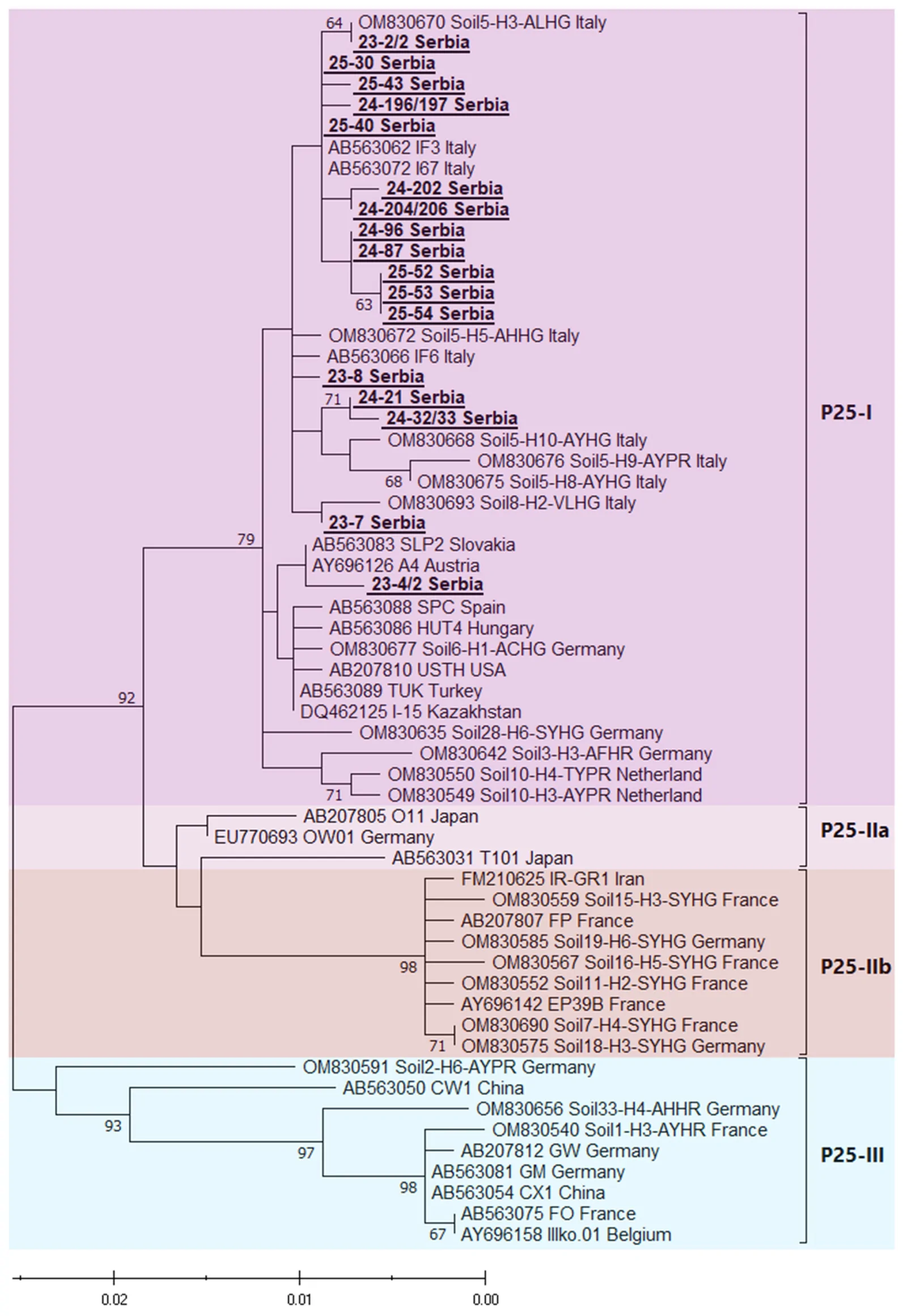
A maximum likelihood phylogenetic tree based on sequence of the *p25* gene constructed by MEGA X with HKY + G as the best-fit nucleotide substitution model. Branch support was assessed by bootstrap analysis with 1000 replicates, and bootstrap values ≥ 70% are shown next to the corresponding nodes. The clusters are highlighted in different background colors: pale lavender (P25-I), pale lilac (P25-IIa), dusty rose (P25-IIb), and pale blue (P25-III). Serbian isolates obtained in this study are shown in bold and underlined. Scale bar represents the number of nucleotide changes per sequence.

Phylogenetic relationships among the *p25* sequences were inferred using the maximum likelihood method with HKY + G as the best-fit nucleotide substitution model. The sequences generated in this study, aligned with representative p25sequences retrieved from GenBank (Supplementary Table S1), resolved into three major phylogenetic groups (P25-I, P25-II, and P25-III), corresponding to the A-, P-, and B-type BNYVV populations, respectively (Figure 4).

All Serbian isolates clustered within the P25-I group, which predominantly comprises European A-type isolates exhibiting diverse tetrad variants. The majority of the Serbian isolates formed a distinct subcluster with Italian isolates, whereas isolate 23-4/2 clustered more closely with isolates from Slovakia and Austria.

**Figure 5:**
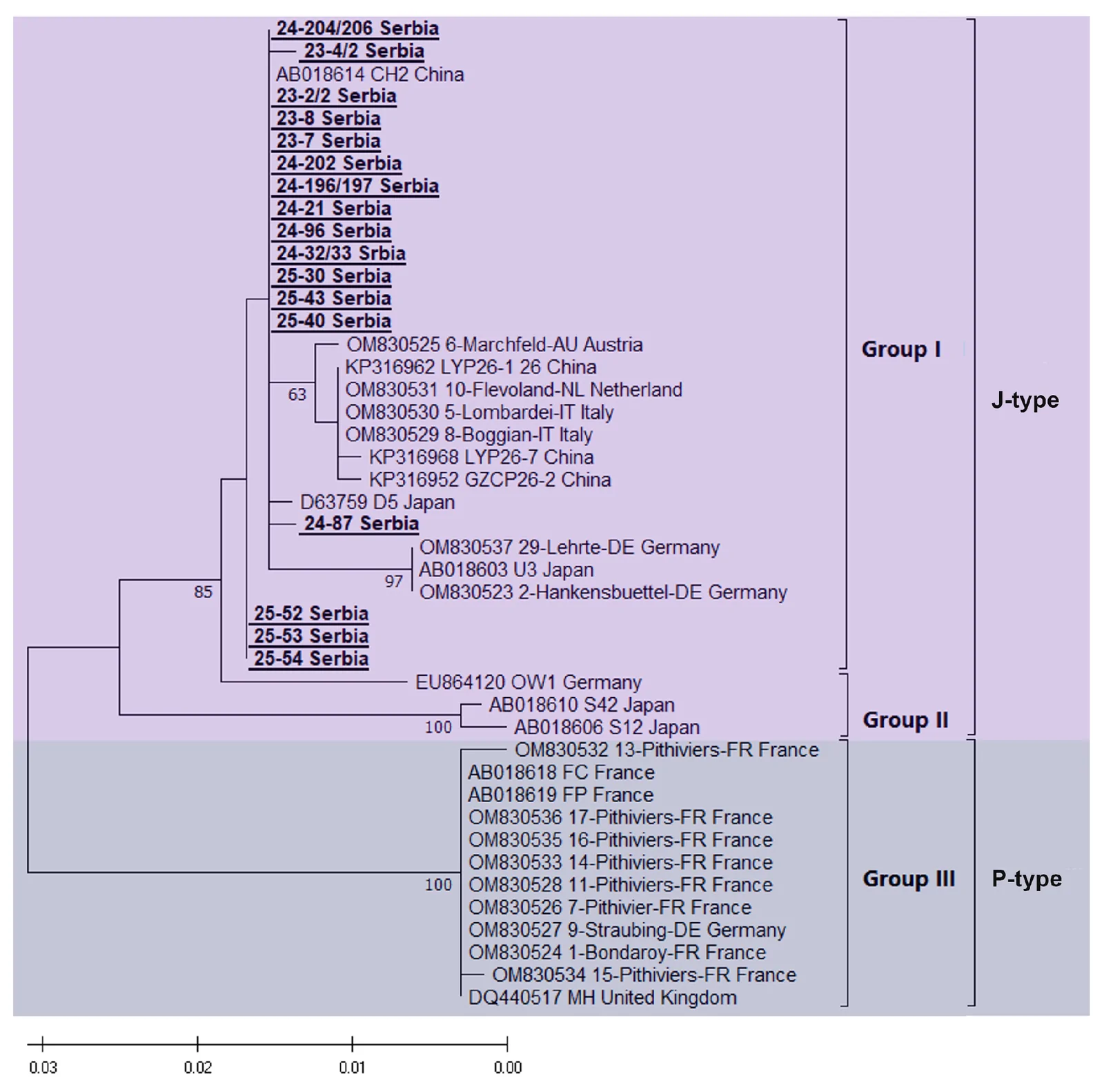
A maximum likelihood phylogenetic tree based on sequence of the *p26* gene constructed by MEGA X with HKY + G as the best-fit nucleotide substitution model. Branch support was assessed by bootstrap analysis with 1000 replicates, and bootstrap values ≥70% are shown next to the corresponding nodes. The J- and P-type lineages are highlighted in different background colors: lavender (J-type) and gray (P-type). Serbian isolates obtained in this study are shown in bold and underlined. Scale bar represents the number of nucleotide changes per sequence.

The presence of RNA5 was investigated in all 17 selected isolates through amplification and sequencing of the *p26* gene (Table 2). RNA5 was detected in all selected Serbian isolates showing the widespread presence of a new type of virus in sugar beet fields. Amplification with the BNYVV-5F1/BNYVV-5R1 primer pair produced amplicons of the expected size in all samples (Supplementary Figure S1), indicating a high prevalence of RNA5 among Serbian BNYVV populations. The RNA5 sequences obtained showed high nucleotide and amino acid sequence similarity, ranging from 99.7% to 100% and 99.5% to 100%, respectively. Based on maximum-likelihood phylogenetic analysis, all Serbian RNA5 p26 sequences clustered within Group I, corresponding to the Asian J-type lineage (Figure 5). Notably, this is the first report of the RNA5 J-type lineage in Serbia, extending the known geographical distribution of this lineage and documenting its presence in Serbian sugar beet production for the first time.

## 4. Discussion

This study provides new insights into the genetic diversity and population structure of BNYVV in Serbia, with a particular emphasis on P25 tetrad variability and the widespread occurrence of RNA5. Furthermore, this work sheds light, for the first time, on the phylogenetic relationships and connectivity between Serbian isolates and global BNYVV populations. BNYVV was detected by ELISA in 88 sugar beet samples collected from 13 of the 31 monitored localities, corresponding to an overall detection rate of 26.5%. Of the 88 ELISA-positive samples, 60 originated from cultivars carrying the *Rz1* resistance gene, whereas 28 were obtained from cultivars carrying the combined *Rz1*+*Rz2* resistance genes. The high prevalence of BNYVV detected in this study is consistent with the long-term presence of rhizomania in Serbia. The disease was first reported in the former Yugoslavia in 1977 in the Srem region [35] and subsequently spread to Banat, Pomoravlje, and Bačka [36]. Since then, rhizomania has become established throughout the major sugar beet-growing regions, resulting in the widespread adoption of resistant sugar beet cultivars as the primary disease management strategy. Although resistant cultivars effectively reduce disease severity and yield losses, their extensive and prolonged use has led to strong selection pressure on the virus population, favoring the emergence and maintenance of RB isolates. The identification of RB isolates carrying altered P25 tetrad, together with the presence of RNA5, supports this hypothesis and suggests that these molecular features may contribute to the adaptation of BNYVV populations.

Despite the high nucleotide sequence similarity observed among Serbian *p25* gene sequences, considerable diversity was detected at the amino acid level within the tetrad region. Our results revealed the presence of multiple P25 tetrad variants, including variants previously reported to be associated with either non-resistance-breaking or resistance-breaking phenotypes. The identification of five distinct tetrad variants within the P25 protein indicates the coexistence of genetically diverse BNYVV populations in Serbian sugar beet-growing regions. This level of diversification aligns with observations in other European production areas. Similarly, Liebe and Varrelmann [21] identified 105 haplotypes comprising 14 different P25 tetrad variants across European BNYVV populations, while Galein et al. [5] documented the simultaneous occurrence of A-, B-, and P-type BNYVV isolates and detected 21 tetrad variants at amino acid positions 67–70 within a single sugar beet production area in Pithiviers, France.

Among the variants detected in the present study, ALHG was the predominant tetrad, whereas AHHG, TLHG, AFHG, and VHHG occurred at lower frequencies, further supporting the ongoing diversification of local viral populations. Previous studies have demonstrated extensive sequence variability at amino acid positions 67–70 of the P25 pathogenicity factor and have linked specific tetrad variants to the ability to overcome host resistance [10,19,25,26]. Chiba et al. [19] suggested that substitutions from alanine to valine at position 67 and from tyrosine to cysteine, histidine, leucine, or glutamine at position 68 are associated with *Rz1*-resistance-breaking isolates. However, to date, resistance-breaking activity has been experimentally validated through reverse genetics approaches for only a limited number of tetrads, including VHHG detected in this study [37]. Nevertheless, since no biological resistance-breaking assay was performed in the present study, this isolate should be considered potentially RB-associated rather than a confirmed resistance-breaking isolate. These findings highlight the importance of continued molecular surveillance to enable early detection of associated resistance-breaking variants and to support sustainable resistance management strategies in Serbian sugar beet production.

Interestingly, in addition to its detection in *Rz1* resistant sugar beet cultivars, the BNYVV RNA5 J-type was also identified in a sugar beet cultivar carrying the combined *Rz1*+*Rz2* resistance genes. Furthermore, differences in the P25 tetrad composition between isolates 24/21 and 24/96, both collected from the same *Rz1*+*Rz2* resistant cultivar in the same field during 2024 but at different sampling times, indicate that the BNYVV population within a single field is dynamic rather than homogeneous. This observation may reflect temporal shifts in the relative abundance of viral variants or the coexistence of multiple viral genotypes within the same field. The detection of the RNA5 J-type in an *Rz1*+*Rz2* resistant cultivar is particularly noteworthy, as no BNYVV populations capable of overcoming Rz2-mediated resistance have been reported to date. Although the presence of the virus alone does not demonstrate that *Rz2* resistance has been overcome, it supports previous observations that combined *Rz1*+*Rz2* resistance does not necessarily provide complete protection against BNYVV under natural field conditions [5,6] and high pressure of virus containing RNA5 in sugar beet fields. Together with the detection of the RNA5 J-type and the observed differences in tetrad composition between samples collected from the same field with *Rz1*+*Rz2* resistant cultivars, these findings indicate that genetically diverse BNYVV populations can persist within a single field, even in cultivars carrying stacked resistance genes.

Phylogenetic analysis of p25 sequences confirmed that all Serbian isolates clustered within the P25-I group, corresponding to A-type virus (Italy strain; A-group *CP* and I-group p25). This group was first found in Italy in the 1950s and spread within Europe, to the Middle East, and to the United States [15,17,19]. The close relationship of Serbian isolates with Italian sequences suggests possible regional connectivity, although the mechanisms underlying this genetic relationship cannot be inferred from the present data. In contrast, the distinct clustering of one Serbian isolate with sequences from Slovakia and Austria may reflect independent introduction events or multiple sources of viral entry into the region.

The presence of the RNA5 segment is also a factor influencing the epidemiology and pathogenicity of BNYVV. Previous research has shown that P-type RNA5 is associated with enhanced virus accumulation in the rootlets of *Rz1*-resistant plants, suggesting that RNA5 is major driver of *Rz1*-resistance breaking by P-type isolates [38], while similar effects have also been reported for J-type RNA5 [6,30]. In the present study, RNA5 was detected in 17 selected Serbian isolates, indicating its widespread occurrence within Serbian BNYVV populations and suggesting that RNA5 may play an important role in their adaptation and resistance-breaking potential. Presence is detected in both Rz1 and *Rz1*+*Rz2* resistant cultivars.

Sequence analysis revealed relatively low genetic diversity among Serbian RNA5 isolates, and phylogenetic analysis clustered all obtained sequences within the J-type. Although the J-type RNA5 was initially considered to be restricted primarily to Asian BNYVV populations, more recent studies have demonstrated a much broader geographical distribution. Several European A-type BNYVV populations have been shown to harbor RNA5 variants belonging to the Asian J-type. In addition to Germany [17], the J-type RNA5 has also been reported in Austria, Italy, and the Netherlands [21]. Furthermore, J-type BNYVV strains are widespread in the major sugar beet-growing regions of Turkey [20].

It has been proposed that the ancestral BNYVV genome, originally consisting of five RNA segments, evolved in native hosts in East Asia before spreading globally to sugar beet production areas [19]. Moreover, the exclusive association of J-type RNA5 with A-type BNYVV may support the hypothesis of historical virus movement from Asia to Europe, considering that A-type isolates are predominant in both regions [21].

## 5. Conclusions

The co-occurrence of diverse P25 tetrad variants and the widespread presence of RNA5 indicate that Serbian BNYVV populations are genetically heterogeneous and evolutionarily dynamic. However, the assessment of population diversity in the present study was based on natural virus populations infecting resistant sugar beet cultivars (*Rz1* and *Rz1*+*Rz2*). During the three-year survey period conducted across the main sugar beet-growing region, BNYVV was detected with an overall incidence of 26.5%. The Asian J-type lineage was detected in all tested Serbian RNA5 p26 sequences, providing the first evidence of its occurrence in Serbia. Future studies should incorporate more systematic soil sampling combined with greenhouse bait plant assays, a larger number of isolates, and amplicon cloning instead of direct sequencing. Such approaches would enable a more accurate evaluation of whether the observed levels of genetic diversity and the frequencies of P25 tetrad variants truly reflect the population structure of BNYVV in Serbia. Furthermore, the detection of variants associated with *Rz1*-resistance breaking, together with the widespread occurrence of RNA5 and positive BNYVV samples collected from *Rz1*+*Rz2* resistant cultivars highlights the need for integrated disease management strategies and continuous molecular surveillance of BNYVV populations to mitigate the risk of resistance breakdown in sugar beet production.

## Data Availability

The original contributions presented in this study are included in the article/Supplementary Material. Further inquiries can be directed to the corresponding authors.

## Supplementary Materials

The following supporting information can be downloaded at: https://www.mdpi.com/article/10.3390/microorganisms14092030/s1, Table S1: Beet necrotic yellow vein virus isolates used for P25 phylogenetic analyses; Table S2: Beet necrotic yellow vein virus isolates used for P26 phylogenetic analyses. Figure S1: Agarose gel electrophoresis of RT-PCR products obtained using primer pairs specific for the *p25* and *p26* genes of beet necrotic yellow vein virus. (A) Amplification of the *p25* gene. (B) Amplification of the *p26* gene. M, molecular weight marker; lanes 1–17, Serbian virus isolates; N, negative control.
