# Supplementary material for: Genetic Diversity of Beet Necrotic Yellow Vein Virus in Serbia: First Detection of J-Type RNA5 and P25 Tetrad Associated with Resistance-Breaking Isolates
Source: Microorganisms. 2026 Sep 11;14(9):2030. doi: 10.3390/microorganisms14092030 (PMC13609331; doi:10.3390/microorganisms14092030)
Supplement: Supplementary file 1 [file microorganisms-14-02030-s001.zip › microorganisms-4489726-supplementary.pdf]

Supplementary Material

Supplementary Table S1. Beet nectrotic yellow vein virus isolates used for P25 phyogenetic analyses

Isolate	Country	GenBank database accession number
IF3	Italy	AB563062
I67		AB563072
IF6		AB563066
Soil5-H3-ALHG		OM830670
Soil5-H5-AHHG		OM830672
Soil5-H8-AYHG		OM830675
Soil5-H9-AYPR		OM830676
Soil5-H10-AYHG		OM830668
Soil8-H2-VLHG		OM830693
SLP2	Slovakia	AB563083
A4	Austria	AY696126
SPC	Spain	AB563088
HUT4	Hungary	AB563086
Soil6-H1-ACHG	Germany	OM830677
Soil28-H6-SYHG		OM830635
Soil3-H3-AFHR		OM830642
Soil19-H6-SYHG		OM830585
Soil18-H3-SYHG		OM830575
Soil2-H6-AYPR		OM830591
Soil33-H4-AHHR		OM830656
OW01		EU770693
GW		AB207812
GM		AB563081
USTH	USA	AB207810
TUK	Turkey	AB563089
I-15	Kazakhstan	DQ462125
Soil10-H4-TYPR	Netherland	OM830550
Soil10-H3-AYPR		OM830549
Soil1-H3-AYHR	France	OM830540
Soil7-H4-SYHG		OM830690
Soil11-H2-SYHG		OM830552
Soil15-H3-SYHG		OM830559
Soil16-H5-SYHG		OM830567
FP		AB207807
EP39B		AY696142
FO		AB563075
Illko.01	Belgium	AY696158
O11	Japan	AB207805
T101		AB563031
IR-GR1	Iran	FM210625
CW1	China	AB563050
CX1		AB563054

Supplementary Table S2. Beet nectrotic yellow vein virus isolates used for P26 phyogenetic analyses

Isolate	Country	GenBank database accession number
5-Lombardei-IT	Italy	OM830530
8-Boggian-IT		OM830529
6-Marchfeld-AU	Austria	OM830525
10-Flevoland-NL	Netherland	OM830531
29-Lehrte-DE	Germany	OM830537
2-Hankensbuettel-DE		OM830523
9-Straubing-DE		OM830527
OW1		EU864120
MH	United Kingdom	DQ440517
13-Pithiviers-FR	France	OM830532
FC		AB018618
FP		AB018619
17-Pithiviers-FR		OM830536
16-Pithiviers-FR		OM830535
15-Pithiviers-FR		OM830534
14-Pithiviers-FR		OM830533
11-Pithiviers-FR		OM830528
7-Pithivier-FR		OM830526
1-Bondaroy-FR		OM830524
S12	Japan	AB018606
S42		AB018610
U3		AB018603
D5		D63759
CH2	China	AB018614
LYP26-1 26		KP316962
LYP26-7		KP316968
GZCP26-2		KP316952

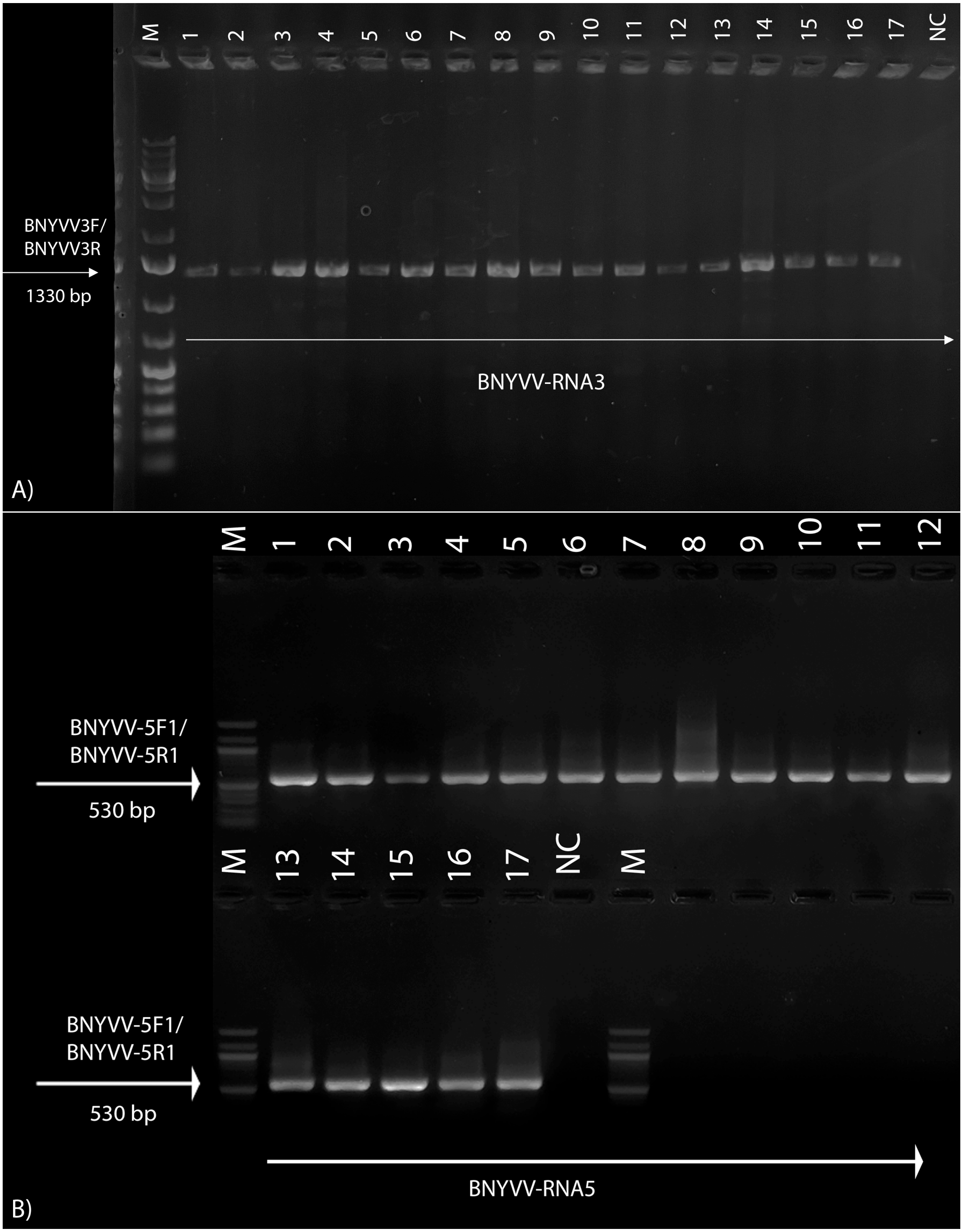

Supplementary Figure S1. Agarose gel electrophoresis of RT-PCR products obtained using primer pairs specific for the *p25* and *p26* genes of beet necrotic yellow vein virus. (A) Amplification of the *p25* gene. (B) Amplification of the *p26* gene. M, molecular weight marker; lanes 1–17, Serbian virus isolates; N, negative control.
